# *catena*-Poly[copper(I)-bis­[μ-3-(1*H*-imidazol-2-yl)pyridine]-copper(I)-di-μ-iodido]

**DOI:** 10.1107/S1600536811033605

**Published:** 2011-08-27

**Authors:** Qing-Guang Zhan

**Affiliations:** a School of Chemistry and Environment, South China Normal University, Guangzhou 510006, People’s Republic of China, and, South China Normal University, Key Laboratory of Technology in Electrochemical Energy Storage and Power Generation in Guangdong Universities, Guangzhou 510006, People’s Republic of China

## Abstract

The title polymeric compound, [Cu_2_I_2_(C_8_H_7_N_3_)_2_]_*n*_ [C_8_H_7_N_3_ = 3-(1*H*-imidazol-2-yl)pyridine (HIPy), where HIPy comes from the *in situ* deca­rboxylation of 2-(pyridin-3-yl)-1*H*-imidazole-4,5-dicarb­oxy­lic acid (H_3_PyIDC)], was obtained under solvo­thermal conditions. Each Cu^I^ cation is in a distorted tetra­hedral coordination environment defined by two iodide anions and two nitro­gen atoms from two individual HIPy ligands. Two Cu^I^ atoms are connected by two HIPy ligands to form a dimer and these dimers are further bridged through the iodide atoms, leading to a chain structure extending parallel to [100]. Moreover, inter­molecular N—H⋯I hydrogen bonds and weak π–π stacking inter­actions [centroid⋯centroid distances of 3.809 (4) Å, an inter­planar separation of 3.345 (3) Å and a ring slippage of 1.822 Å] between pyridyl rings link the chains into a two-dimensional supra­molecular network in the *ac* plane.

## Related literature

For general background on the deca­rboxylation of *N*-heterocyclic carb­oxy­lic acid ligands, see: Chen & Tong (2007 ▶); Sun *et al.* (2006 ▶); Yigit *et al.* (2006 ▶); Zhong *et al.* (2010 ▶).
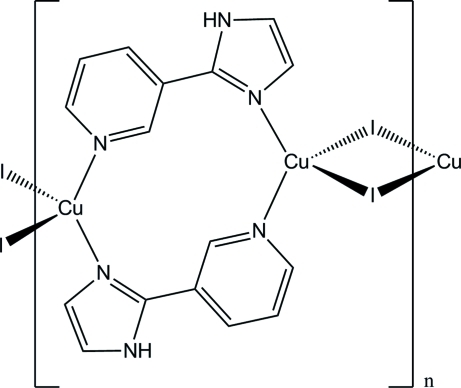


## Experimental

### 

#### Crystal data

[Cu_2_I_2_(C_8_H_7_N_3_)_2_]
*M* *_r_* = 671.22
Triclinic, 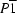

*a* = 8.141 (3) Å
*b* = 8.306 (3) Å
*c* = 8.816 (5) Å
α = 114.683 (6)°
β = 101.989 (5)°
γ = 108.258 (4)°
*V* = 473.1 (4) Å^3^
*Z* = 1
Mo *K*α radiation
μ = 5.52 mm^−1^
*T* = 298 K
0.35 × 0.32 × 0.30 mm

#### Data collection

Bruker SMART APEXII CCD area-detector diffractometer
Absorption correction: multi-scan (*APEX2*; Bruker, 2004 ▶) *T*_min_ = 0.248, *T*_max_ = 0.288
2452 measured reflections
1682 independent reflections
1506 reflections with *I* > 2σ(*I*)
*R*_int_ = 0.018

#### Refinement

*R*[*F* ^2^ > 2σ(*F* ^2^)] = 0.031
*wR*(*F* ^2^) = 0.080
*S* = 1.06
1682 reflections
118 parameters
H-atom parameters constrained
Δρ_max_ = 0.86 e Å^−3^
Δρ_min_ = −0.69 e Å^−3^

### 

Data collection: *APEX2* (Bruker, 2004 ▶); cell refinement: *APEX2*; data reduction: *APEX2*; program(s) used to solve structure: *SHELXS97* (Sheldrick, 2008 ▶); program(s) used to refine structure: *SHELXL97* (Sheldrick, 2008 ▶); molecular graphics: *ORTEPIII* (Burnett & Johnson, 1996 ▶); *PLATON* (Spek, 2009 ▶); software used to prepare material for publication: *SHELXL97*.

## Supplementary Material

Crystal structure: contains datablock(s) I, global. DOI: 10.1107/S1600536811033605/zl2401sup1.cif

Structure factors: contains datablock(s) I. DOI: 10.1107/S1600536811033605/zl2401Isup2.hkl

Additional supplementary materials:  crystallographic information; 3D view; checkCIF report

## Figures and Tables

**Table 1:** Hydrogen-bond geometry (Å, °)

*D*—H⋯*A*	*D*—H	H⋯*A*	*D*⋯*A*	*D*—H⋯*A*
N2—H2⋯I1^i^	0.86	2.83	3.588 (5)	148

Symmetry code: (i) 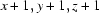.

**Fig. 1.**
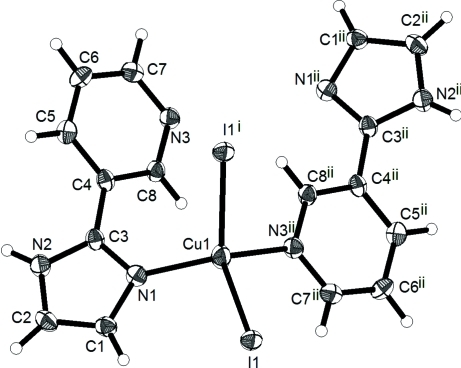
The molecular structure of the title compound, showing the atom-labelling scheme and displacement ellipsoids drawn at the 30% probability level. Symmetry codes: (i) -x-1, -y+1, -z; (ii) -x, -y+1, -z.

**Fig. 2.**
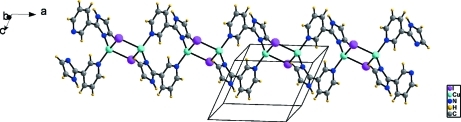
The crystal packing of the title compound, showing the one-dimensional chain structure extending parallel to [100].

**Fig. 3.**
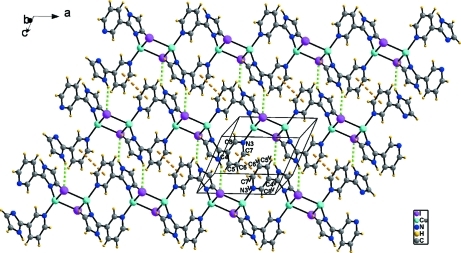
A view showing part of the two-dimensional supramolecular network linked by N–H···I hydrogen bonds and weak π-π stacking interactions. Hydrogen bonds and π-π stacking interactions are shown as dashed lines. Symmetry code: (v) 1-x, 2-y, 1-z).

## supplementary crystallographic information

### Comment

In recent years, *in situ* metal/ligand reactions have been widely investigated for the discovery of new organic reactions, elucidation of reaction mechanisms, as well as generation of novel coordination polymers (Chen *et al.*, 2007). Among them, hydrothermal decarboxylation of *N*-heterocyclic carboxylic acid ligands has been shown to occur in the presence of metal ions (Sun *et al.*, 2006; Yigit *et al.*, 2006; Zhong *et al.*, 2010). For example, Sun *et al.* (2006) have synthesized two lanthanide sulfate–carboxylates, [Ln(HIMC)(SO_4_)(H_2_O)] (Ln = Dy and Eu, HIMC = 4-imidazolecarboxylic acid ), by using *in situ* decarboxylation of 4,5-imidazoledicarboxylic acid in the presence of Cu(II) ions. However, as far as we know, no decarboxylation based on 2-(pyridin-3-yl)-1H-imidazole-4,5-dicarboxylic acid (H_3_PyIDC) under solvothermal reaction conditions has been documented. In this work, we report the synthesis and structure of the polymeric title complex, using H_3_PyIDC as one of the starting materials. Under the solvothermal reaction conditions and in the presence of CuI decarboxylation occurs and H_3_PyIDC is transformed into HIPy which is incorporated into the polymeric title complex.

The asymmmetric unit of the title compound contains one Cu^+^ cation, one I^-^ anion and one HIPy neutral ligand. As shown in Fig. 1, the Cu^I^ cation exhibits a distorted tetrahedral coordination, made up of two iodide anions and two nitrogen atoms from two individual HIPy ligands. The Cu···I bond lengths are 2.7331 (12) and 2.7887 (14) Å.

In the crystal structure, two Cu^I^ atoms are connected through two HIPy ligands via their N_imidazole_ and N_pyridyl_ atoms to form a dimer with a Cu···Cu separation of 5.3621 (21) Å; these dimers are further bridged through the *µ*_2_-I atoms, leading to a 1D chain structure extending parallel to [100] (Fig. 2). Intermolecular N2–H2···I1^iii^ hydrogen bonds between the imidazole N-H groups and the I atoms (Table 1) [symmetry code: (iii) x+1, y+1, z+1] link the chains into a 2D supramolecular network in the *ac* plane (Fig. 3). The crystal structure is further stabilized by weak slipped π-π stacking interactions between neighbouring pyridyl rings (N3/C4-C8 and N3^v^/C4^v^-C8^v^, symmetry code: (v) 1-x, 2-y, 1-z), with centroid···centroid distances of 3.809 (4) Å, an interplanar separation of 3.345 (3) Å and a ring slippage of 1.822 Å (Fig. 3).

### Experimental

A mixture of H_3_PyIDC (46.6 mg, 0.2 mmol), CuI (38.1 mg, 0.2 mmol), 8 mL EtOH/H_2_O (1:1, v/v), and 0.1 mL Et_3_N was sealed in a 15mL Teflon-lined stainless steel autoclave, heated at 443 K for 48 h, and then slowly cooled to room temperature at a rate of 273 K/h. Yellow block-shaped crystals of the title compound were isolated, washed with distilled water, and dried in air (yield: 25%). Anal. Calcd. for C_8_H_7_CuIN_3_: C, 28.63, H, 2.10, N, 12.52. Found: C, 28.73, H, 2.05, N, 12.48%.

### Refinement

All non-hydrogen atoms were assigned anisotropic displacement parameters in the refinement. All hydrogen atoms were positioned geometrically and refined using a riding model, with C—H = 0.93 Å and N—H = 0.86 Å and with *U*_iso_(H) = 1.2 *U*_eq_(C, N).

### Crystal data

**Table:**

[Cu_2_I_2_(C_8_H_7_N_3_)_2_]	*Z* = 1
*M**_r_* = 671.22	*F*(000) = 316
Triclinic, *P*1	*D*_x_ = 2.356 Mg m^−^^3^
Hall symbol: -P 1	Mo *K*α radiation, λ = 0.71073 Å
*a* = 8.141 (3) Å	Cell parameters from 1252 reflections
*b* = 8.306 (3) Å	θ = 2.8–26.8°
*c* = 8.816 (5) Å	µ = 5.52 mm^−^^1^
α = 114.683 (6)°	*T* = 298 K
β = 101.989 (5)°	Block, yellow
γ = 108.258 (4)°	0.35 × 0.32 × 0.30 mm
*V* = 473.1 (4) Å^3^	

### Data collection

**Table:**

Bruker SMART APEXII CCD area-detector diffractometer	1682 independent reflections
Radiation source: fine-focus sealed tube	1506 reflections with *I* > 2σ(*I*)
graphite	*R*_int_ = 0.018
φ and ω scans	θ_max_ = 25.2°, θ_min_ = 2.8°
Absorption correction: multi-scan (*APEX2*; Bruker, 2004)	*h* = −8→9
*T*_min_ = 0.248, *T*_max_ = 0.288	*k* = −9→5
2452 measured reflections	*l* = −10→10

### Refinement

**Table:**

Refinement on *F*^2^	Primary atom site location: structure-invariant direct methods
Least-squares matrix: full	Secondary atom site location: difference Fourier map
*R*[*F*^2^ > 2σ(*F*^2^)] = 0.031	Hydrogen site location: inferred from neighbouring sites
*wR*(*F*^2^) = 0.080	H-atom parameters constrained
*S* = 1.06	*w* = 1/[σ^2^(*F*_o_^2^) + (0.0418*P*)^2^ + 0.3135*P*] where *P* = (*F*_o_^2^ + 2*F*_c_^2^)/3
1682 reflections	(Δ/σ)_max_ = 0.001
118 parameters	Δρ_max_ = 0.86 e Å^−^^3^
0 restraints	Δρ_min_ = −0.69 e Å^−^^3^

### Special details

**Table:**

Geometry. All esds (except the esd in the dihedral angle between two l.s. planes) are estimated using the full covariance matrix. The cell esds are taken into account individually in the estimation of esds in distances, angles and torsion angles; correlations between esds in cell parameters are only used when they are defined by crystal symmetry. An approximate (isotropic) treatment of cell esds is used for estimating esds involving l.s. planes.
Refinement. Refinement of F^2^ against ALL reflections. The weighted R-factor wR and goodness of fit S are based on F^2^, conventional R-factors R are based on F, with F set to zero for negative F^2^. The threshold expression of F^2^ > 2sigma(F^2^) is used only for calculating R-factors(gt) etc. and is not relevant to the choice of reflections for refinement. R-factors based on F^2^ are statistically about twice as large as those based on F, and R- factors based on ALL data will be even larger.

### Fractional atomic coordinates and isotropic or equivalent isotropic displacement parameters (Å^2^)

**Table:**

	*x*	*y*	*z*	*U*_iso_*/*U*_eq_	
I1	−0.63541 (5)	0.55664 (5)	−0.19170 (4)	0.03979 (15)	
Cu1	−0.29230 (10)	0.58503 (11)	−0.03571 (10)	0.0494 (2)	
N1	−0.1172 (6)	0.8746 (7)	0.1128 (6)	0.0359 (10)	
N2	0.0984 (6)	1.1683 (7)	0.3412 (6)	0.0410 (10)	
H2	0.1990	1.2584	0.4362	0.049*	
C5	0.2418 (7)	0.9476 (8)	0.4986 (7)	0.0387 (12)	
H5	0.2385	1.0553	0.5891	0.046*	
C6	0.3354 (7)	0.8513 (9)	0.5428 (7)	0.0398 (12)	
H6	0.3934	0.8906	0.6634	0.048*	
C7	0.3418 (7)	0.6968 (8)	0.4067 (7)	0.0379 (12)	
H7	0.4039	0.6317	0.4384	0.045*	
C3	0.0440 (7)	0.9714 (7)	0.2574 (7)	0.0329 (11)	
C1	−0.1648 (7)	1.0191 (8)	0.1087 (7)	0.0371 (12)	
H1	−0.2713	0.9951	0.0220	0.044*	
C2	−0.0336 (8)	1.2007 (8)	0.2495 (7)	0.0414 (13)	
H2A	−0.0330	1.3221	0.2781	0.050*	
C4	0.1521 (7)	0.8819 (7)	0.3165 (7)	0.0324 (11)	
C8	0.1698 (7)	0.7266 (7)	0.1884 (7)	0.0343 (11)	
H8	0.1135	0.6846	0.0668	0.041*	
N3	0.2635 (6)	0.6336 (6)	0.2299 (6)	0.0364 (10)	

### Atomic displacement parameters (Å^2^)

**Table:**

	*U*^11^	*U*^22^	*U*^33^	*U*^12^	*U*^13^	*U*^23^
I1	0.0394 (2)	0.0415 (2)	0.0382 (2)	0.02226 (17)	0.01033 (16)	0.01990 (18)
Cu1	0.0506 (4)	0.0349 (4)	0.0442 (4)	0.0235 (3)	0.0051 (3)	0.0086 (3)
N1	0.035 (2)	0.038 (3)	0.037 (2)	0.020 (2)	0.0167 (19)	0.018 (2)
N2	0.039 (2)	0.034 (3)	0.042 (3)	0.015 (2)	0.013 (2)	0.016 (2)
C5	0.036 (3)	0.039 (3)	0.038 (3)	0.016 (2)	0.018 (2)	0.016 (3)
C6	0.039 (3)	0.051 (3)	0.030 (3)	0.021 (3)	0.014 (2)	0.020 (3)
C7	0.034 (3)	0.040 (3)	0.045 (3)	0.017 (2)	0.016 (2)	0.026 (3)
C3	0.033 (3)	0.030 (3)	0.035 (3)	0.016 (2)	0.015 (2)	0.014 (2)
C1	0.039 (3)	0.039 (3)	0.040 (3)	0.023 (3)	0.017 (2)	0.022 (3)
C2	0.053 (3)	0.036 (3)	0.050 (3)	0.028 (3)	0.026 (3)	0.025 (3)
C4	0.030 (2)	0.027 (3)	0.037 (3)	0.013 (2)	0.015 (2)	0.013 (2)
C8	0.033 (3)	0.032 (3)	0.033 (3)	0.018 (2)	0.011 (2)	0.011 (2)
N3	0.033 (2)	0.034 (2)	0.039 (2)	0.017 (2)	0.0118 (19)	0.016 (2)

### Geometric parameters (Å, °)

**Table:**

I1—Cu1	2.7331 (12)	C6—C7	1.368 (8)
I1—Cu1^i^	2.7887 (14)	C6—H6	0.9300
Cu1—N1	1.994 (4)	C7—N3	1.345 (7)
Cu1—N3^ii^	2.030 (4)	C7—H7	0.9300
Cu1—I1^i^	2.7887 (14)	C3—C4	1.464 (7)
N1—C3	1.335 (6)	C1—C2	1.353 (7)
N1—C1	1.384 (7)	C1—H1	0.9300
N2—C3	1.348 (7)	C2—H2A	0.9300
N2—C2	1.372 (7)	C4—C8	1.388 (7)
N2—H2	0.8600	C8—N3	1.342 (6)
C5—C6	1.376 (8)	C8—H8	0.9300
C5—C4	1.392 (7)	N3—Cu1^ii^	2.030 (4)
C5—H5	0.9300		
			
Cu1—I1—Cu1^i^	79.47 (3)	N3—C7—H7	118.1
N1—Cu1—N3^ii^	127.88 (17)	C6—C7—H7	118.1
N1—Cu1—I1	105.56 (12)	N1—C3—N2	110.0 (4)
N3^ii^—Cu1—I1	106.58 (12)	N1—C3—C4	126.1 (5)
N1—Cu1—I1^i^	109.74 (13)	N2—C3—C4	123.8 (4)
N3^ii^—Cu1—I1^i^	103.36 (13)	C2—C1—N1	110.0 (5)
I1—Cu1—I1^i^	100.53 (3)	C2—C1—H1	125.0
C3—N1—C1	105.7 (4)	N1—C1—H1	125.0
C3—N1—Cu1	128.9 (4)	C1—C2—N2	105.7 (5)
C1—N1—Cu1	123.9 (3)	C1—C2—H2A	127.2
C3—N2—C2	108.5 (4)	N2—C2—H2A	127.2
C3—N2—H2	125.7	C8—C4—C5	117.6 (5)
C2—N2—H2	125.7	C8—C4—C3	119.7 (5)
C6—C5—C4	119.1 (5)	C5—C4—C3	122.6 (5)
C6—C5—H5	120.4	N3—C8—C4	123.8 (5)
C4—C5—H5	120.4	N3—C8—H8	118.1
C7—C6—C5	119.0 (5)	C4—C8—H8	118.1
C7—C6—H6	120.5	C8—N3—C7	116.6 (4)
C5—C6—H6	120.5	C8—N3—Cu1^ii^	121.6 (3)
N3—C7—C6	123.8 (5)	C7—N3—Cu1^ii^	121.8 (4)
			
Cu1^i^—I1—Cu1—N1	−114.10 (13)	C3—N1—C1—C2	−0.1 (6)
Cu1^i^—I1—Cu1—N3^ii^	107.49 (14)	Cu1—N1—C1—C2	167.1 (3)
Cu1^i^—I1—Cu1—I1^i^	0.0	N1—C1—C2—N2	0.6 (6)
N3^ii^—Cu1—N1—C3	−78.1 (5)	C3—N2—C2—C1	−1.0 (6)
I1—Cu1—N1—C3	155.6 (4)	C6—C5—C4—C8	−3.0 (7)
I1^i^—Cu1—N1—C3	48.1 (4)	C6—C5—C4—C3	177.8 (5)
N3^ii^—Cu1—N1—C1	117.9 (4)	N1—C3—C4—C8	39.7 (7)
I1—Cu1—N1—C1	−8.4 (4)	N2—C3—C4—C8	−138.2 (5)
I1^i^—Cu1—N1—C1	−115.9 (4)	N1—C3—C4—C5	−141.1 (5)
C4—C5—C6—C7	1.7 (8)	N2—C3—C4—C5	41.0 (7)
C5—C6—C7—N3	0.9 (8)	C5—C4—C8—N3	1.9 (7)
C1—N1—C3—N2	−0.6 (5)	C3—C4—C8—N3	−178.9 (4)
Cu1—N1—C3—N2	−166.8 (3)	C4—C8—N3—C7	0.6 (7)
C1—N1—C3—C4	−178.7 (5)	C4—C8—N3—Cu1^ii^	−177.5 (4)
Cu1—N1—C3—C4	15.0 (7)	C6—C7—N3—C8	−2.1 (7)
C2—N2—C3—N1	1.0 (6)	C6—C7—N3—Cu1^ii^	176.0 (4)
C2—N2—C3—C4	179.2 (5)		

Symmetry codes: (i) −*x*−1, −*y*+1, −*z*; (ii) −*x*, −*y*+1, −*z*.

### Hydrogen-bond geometry (Å, °)

**Table:**

*D*—H···*A*	*D*—H	H···*A*	*D*···*A*	*D*—H···*A*
N2—H2···I1^iii^	0.86	2.83	3.588 (5)	148.

Symmetry codes: (iii) *x*+1, *y*+1, *z*+1.
